# Experimental Simulation of Directional Crystallization of SiMo Cast Iron Alloyed with Al and Cr

**DOI:** 10.3390/ma17112592

**Published:** 2024-05-28

**Authors:** Krzysztof Morgiel, Dariusz Kopyciński

**Affiliations:** Faculty of Foundry Engineering, AGH University of Krakow, al. A. Mickiewicza 30, 30-059 Krakow, Poland; djk@agh.edu.pl

**Keywords:** Fe-C alloys, SiMo cast iron, Al, Cr, directional crystallization, Bridgman–Stockbarger

## Abstract

SiMo ductile cast iron combines ease of part fabrication with good mechanical properties, including a usable plasticity range. Its poor corrosion resistance inherited from grey cast iron could be alleviated through alloying with Al or Cr additions capable of forming a dense oxide scale protecting the substrate. However, the presence of Al and Cr in cast iron tends to make the material brittle, and their optimum alloying additions need to be studied further. The present work was aimed at investigating the effect of crystallization rates on microstructure changes during directional crystallization of SiMo-type alloys with up to 3.5% Al and 2.4% Cr. The experiment was performed using the Bridgman–Stockbarger method. The tubular crucible was transferred from the hot section to cold section at rates ranging from 5 mm/h to 30 mm/h with a 4/5 crucible length and then quenched. The introduced Al promoted graphitization up to a point, wherein, at the highest applied addition, the graphite precipitation preceded crystallization of the rest of the melt. A rising level of Cr in these alloys from 1% to 2.4% resulted in the formation of low and high contents of pearlite, respectively. The higher crystallization rates proved effective in increasing the ferrite content at the expense of pearlite. In the investigated cast iron samples with smaller applied alloying additions, Widmanstätten ferrite or ausferrite, i.e., fine acircular phase, were often found. The switch from directional crystallization to quenching caused a transition from a liquid to solid state, which started with nucleation of islands of fine austenite dendrites with chunky graphite eutectic separating them. As these islands expanded, they pushed alloying additions to their sides, promoting carbide or pearlite formation in these places and forming a super-cell-like structure. The performed experiments helped gather information concerning the sensitivity of the microstructure of SiMo cast iron modified with Al and Cr to crystallization rates prevailing in heavy cast structures.

## 1. Introduction

SiMo ductile cast irons (DCI) belong to a class of materials presenting both high strength and usable plasticity [1]. The increasing amount of Mo from 0.4 wt. % to 0.8 wt. % boosts precipitation of M_6_C carbides at ferrite grain boundaries (simultaneously, Si-stabilizing ferrite has to be added to counteract the development of pearlite) and allows the tensile strength of these alloys to rise from ~400 MPa to ~600 MPa, even as the elongation falls from ~8% to ~4% [1,2,3]. These relatively good plastic properties, being unusual for cast iron, are achieved by graphite spheroidization in the ferritic matrix caused by the addition of Mg [4,5,6]. As in the case of most metallic materials, their tensile strength lowers with the rise in temperature, i.e., in the case of SiMo51, its R_m_ at RT equals ~610 MPa, but at 800 °C, it falls down to ~53 MPa, limiting its application potential. This low-cost material is an alternative for steel, as parts produced from the latter require time and energy-consuming steps covering machining and heat treatment. The SiMo DCI applications cover combustion engine blocks, turbocharger housings, and a range of similar mechanically and thermally loaded parts [1,7].

The corrosion of SiMo DCIs is, in most of their expected applications, unavoidable. It has stimulated extensive research in that area, including the examination of parts made of these alloys subjected to tests by work, i.e., installed in car exhaust manifolds [7,8,9]. These and accompanying laboratory testing showed that oxidizing atmospheres, and especially those rich in water vapor or SO_2_, cause fast growth consisting of iron oxides growing outward and iron–silicon spinel growing inward with a layer of SiO_2_ at the scale/substrate boundary [7,8,9,10,11,12]. The scale weight gain with time follows a roughly parabolic trend typical for out-of-core diffusion-controlled processes, which could eventually limit corrosion-induced degradation. However, the scanning electron microscopy (SEM) observations showed that the scale upper layer was composed of fine whiskers/platelets, while the lower ones were highly brittle and usually cracked. In effect, it will peel off at even low loading exposing the substrate for successive corrosion attacks. Therefore, investigations aimed at assessing to what extent it would be possible to alloy SiMo DCI with Al and Cr forming tight and well-adhering scales—without significantly diminishing the ductility of this material—are needed.

The crystallization of cast iron parts starts and proceeds faster at their near-surface areas only to slow down away from them. In effect, the initially formed fine microstructure is gradually substituted with the coarser columnar grains, growing inward until they reach the center area, filled with homogenously nucleated equiaxed ones [13]. This crystallization process could be followed by sectioning of ingots of various sizes, but increasing the number of investigated alloys makes the task extremely laborious. However, the application of the Bridgman–Stockbarger method enables the crystallization of cast irons to be experimentally simulate, and this approach enables data on more alloy compositions with good control over all involved parameters to be acquired [14]. This experimental set-up relies on melting a sample of alloy in a thin-walled ceramic tube placed in a furnace and then lowering it into a cold container at a chosen rate. As a result, at slow cooling rates, a microstructure characteristic for the columnar grains in heavy ingots is formed, while after quenching, it could represent what is usually formed at the sub-surface of ingots. In the case of SiMo DCI, a disadvantage of this approach is a lack of possibility to retain graphite spheroidizing and inoculant additions, namely Mg and Ca. This is because in such a closed experimental set-up there is no possibility to add anything into cast iron melted in such a small tube as that used as a mold in the present experiment (the standard casting procedure requires the introduction of these elements just before casting). However, other steps, such as nucleation and growth of all involved phases, should properly represent the real casting conditions.

The equilibrium Fe-C phase diagram shows the presence of the eutectic transition at 1154 °C, while in the metastable Fe-Fe_3_C system, it is lowered down to 1148 °C [15]. The latter is significantly affected by the alloying additions, of which P and Si have the greatest impact, i.e., the former lowers it, while the latter causes it to increase [16,17]. Having a wider gap in between both these temperatures at lower cooling rates causes a larger fraction of graphite to be formed in such conditions. The silicon in this type of cast iron also lowers the shrinkage of the castings to the extent that at ~3% of this addition, this negative phenomenon is practically nullified [18]. In DCI, small Mo additions (up to ~1%) are practically neutral in terms of the stability of phase fields in this system. A similar situation is noted for Al, but at low concentrations (<2%), it disrupts spheroidization of graphite. This effect is only slightly alleviated at higher alloying additions [19]. The Cr diminishes eutectic temperature but strongly promotes perlite formation; therefore, it is generally advised to keep its concentration in DCI at a low level (<0.05%) [17,20].

The microstructure of DCI strongly varies depending on the cooling rate. The experiments involving casting SiMo50-7 (C: 2.61%, Si: 5.07%, Mo: 0.7%, Mn: 0.26%, P: 0.035%, S: 0.012%, Mg: 0.037%) into green sand molds with diameters of 3 mm, 6 mm, and 9 mm resulted in evident coarsening of the spheroidal graphite and ferrite grains [21]. Simultaneously, the amount of M_6_C carbides located at original austenite boundaries and surrounding the colonies of perlite remain practically unaffected. This observation was supported later on in an investigation using SiMo45-6 cast into a wedge-shaped mold, which showed that the volume of carbides and pearlite precipitates is more closely related to segregations during solidification than to cooling rates at the eutectoid temperature [22]. The presence of Cr in cast iron generally increases the amount of pearlite and contributes to the formation of M_7_C_3_ carbides [20,23], while additions of up to ~2.5% Al supports graphitization, simultaneously promoting ferrite phase [24]. Starting even from small Al additions, it forms oxides, which contribute to improving the inoculation of the iron melt with (MnX)S compound [25]. The introduction of larger amounts of Al (up to 3%) with a simultaneous reduction in Si down to 2.5% helped to increase the A1 temperature (ferrite to austenite transformation), resulting in the elaboration of SiMo1000 alloy capable of operating even at 860 °C. Unfortunately, higher additions of Si and Al degrade the plastic properties of SiMo DCIs, but understanding the accompanying microstructure changes is currently limited.

The aim of the present work on the SiMo DCI was to describe the effect of the simultaneous introduction of Al and Cr additions (up to 3.5% and 2.5%, respectively) on the microstructure developed under varying cooling rates corresponding to that of heavy castings. The samples were obtained using the Bridgman–Stockbarger method, while observations were performed on their Nital etched metallographic sections using light microscopy (LM).

## 2. Experimental Procedure

### 2.1. Alloys and Crucibles Characteristics

**Figure 1:**
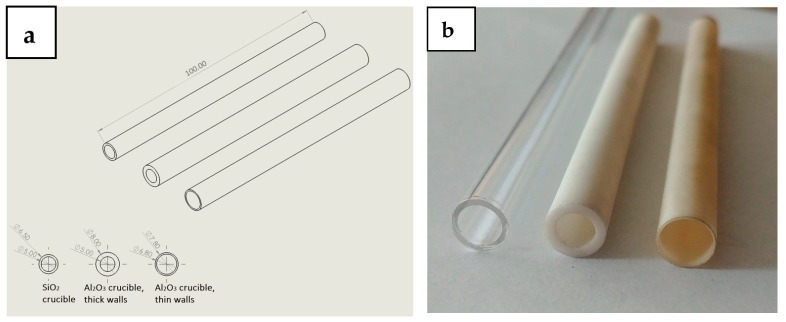
Set of drawings with given dimensions of SiO_2_ and Al_2_O_3_ tubes used as crucibles in direct crystallization experiment (**a**) and their photograph (**b**).

**Figure 2:**
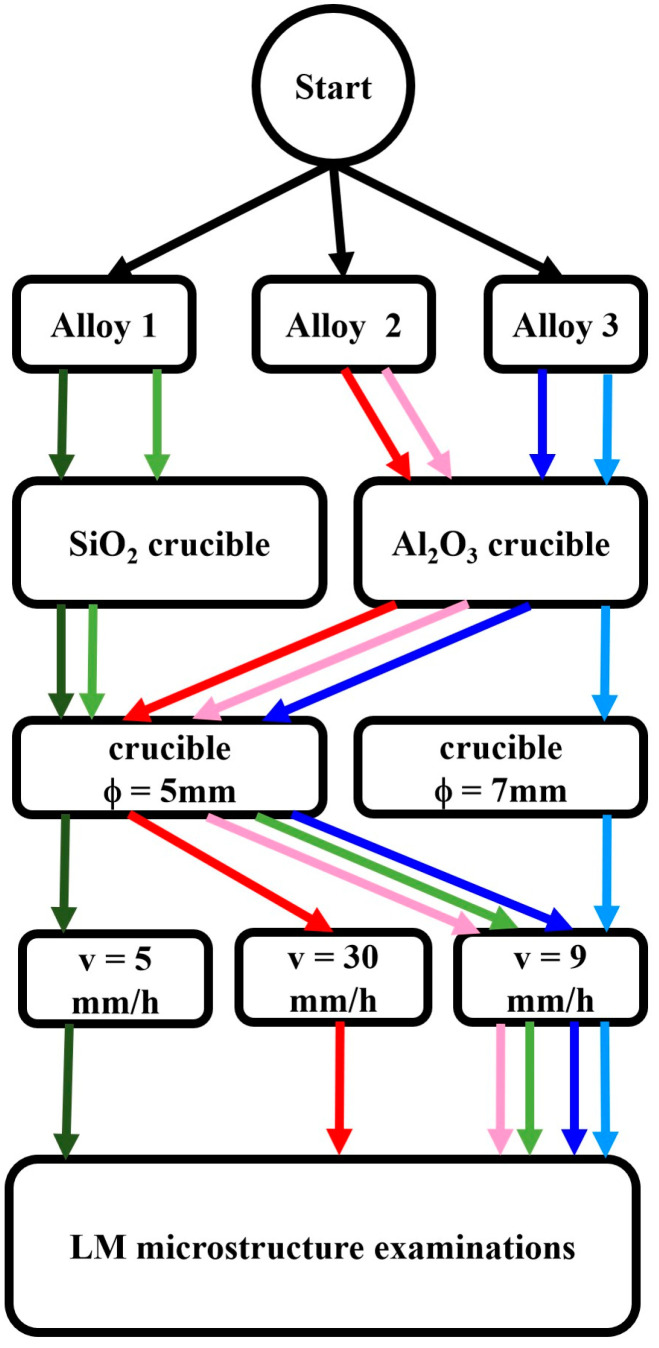
Flow chart presenting experimental procedure (same shades of green, red and blue represent alloys of the same alloy composition, while “v” stands for retraction rate).

Three cast irons with compositions close to SiMo5-10 and differing in raised Al and Cr additions were used in this study (Table 1). The experiment covered their melting in the tubular crucibles (*ϕ* = 5 mm or 7 mm, h = 10 mm) and then slow retraction from the furnace into the liquid gallinstane (Ga 68.5%, In 21.5%, Zn 10%) cooled section of the stand at speeds of from 5 mm/h to 9 mm/h and 30 mm/h. After the lower half of the crucible left the furnace, it was rapidly pushed into the cooled part. The alloys of composition no. 1 were prepared using a SiO_2_ crucible (*ϕ*—5 mm, thin walls), alloys of composition no. 2 were prepared using an Al_2_O_3_ crucible (*ϕ*—5 mm, thick walls), and alloys of composition no. 3 were also prepared with an Al_2_O_3_ crucible (*ϕ*—5 mm and *ϕ*—7 mm with thick and thin walls, respectively) (Figure 1). The experimental procedure is presented in a flow chart in Figure 2. The carbon equivalent (CE) as well as stable (T_st_) and metastable (T_met_) eutectic temperatures were calculated using the following formulas (Table 2):

CE = %C + 0.28%Si + 0.303%P − 0.007%Mn + 0.033%Cr + 0.092%Cu + 0.011%Mo + 0.054%Ni + 0.125 Al(1)

(2) $$S_{c}=\frac{C}{4.26\;-\;0.28\%Si\;-\;0.303\%P+0.007\%Mn\;-\;0.033\%Cr\;-\;0.092\%Cu\;-\;0.011\%Mo\;-\;0.054\%Ni\;-\;0.125\%Al}$$

T_st_ = 1154 °C + 4(%Si) + 4(%Ni) + 8(%Al) − 2(%Mn) − 2(%Mg) (3)

T_met_ = 1148 °C − 15(%Si) − 6(%Ni) − 15(%Al) + 3(%Mn) + 3(%Mg) (4)

### 2.2. Stand for Directional Crystallization and Process Description

**Figure 3:**
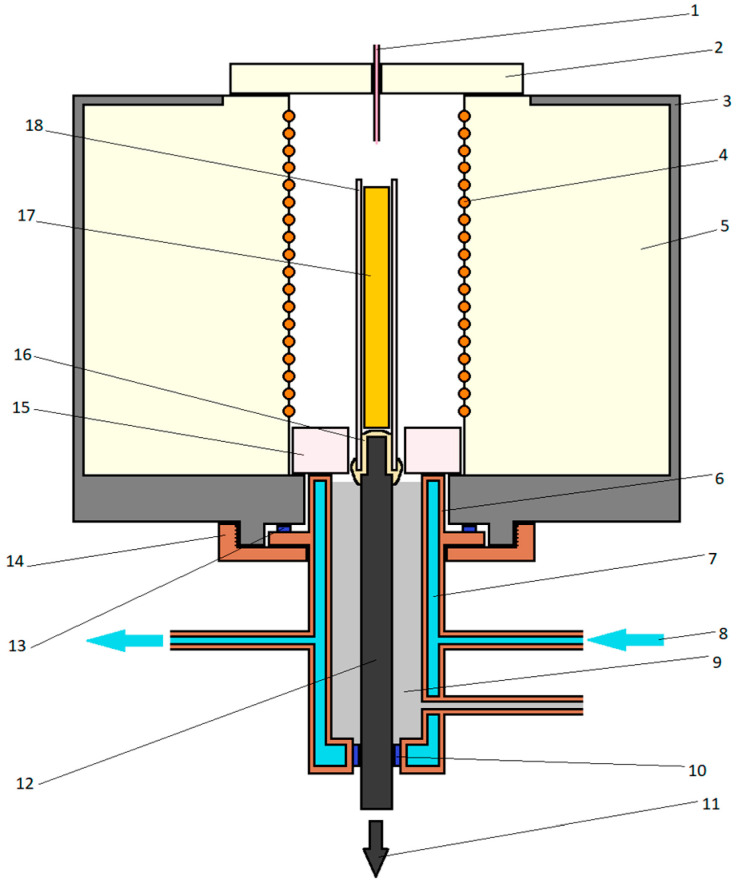
Scheme of “Experimental Furnace for Crystallization”: 1—thermocouple in ceramic cover controlling temperature inside heating chamber, 2—furnace cover, 3—furnace housing, 4—winding, 5—heating chamber lining, 6—copper cooler, 7—water, 8—water flow direction, 9—coolant, 10—gasket no. 1, 11—sample holder travel direction, 12—sample holder, 13—gasket no. 2, 14—mounting nut, 15—crystallizer insulator, 16—crucible fixing glue, 17—sample, 18—crucible.

Melting was performed in the PEKr-1500 furnace dedicated for directional crystallization experiments produced by PUH ENERGOSYSTEM (Skawina, Poland) (Figure 3). It is a stand designed for experiments with the Bridgman–Stockbarger method [12]. Melting of the batch was performed in the electromagnetic induction tube furnace. The directional crystallization was realized in three steps. The first involved heating the crucible with the furnace from ambient temperature to the set temperature (1400 °C) at a rate of 50 °C per minute. The second step covered homogenization of the melt by maintaining it at this temperature for 5 min or 10 min for the thin- and thick-walled Al_2_O_3_ crucibles, respectively. In the third step, the crucible was gradually moved down from the heating chamber into the crystallizer at a set speed. At the moment half of the tubular crucible left the furnace, it was rapidly pushed down into the cold section. The coolant tank made of copper was filled with liquid galinstane and washed from the outside with water.

### 2.3. Microstructure Characterization Details

The obtained ingots were grounded on a sandpaper until longitudinal sections were obtained. Then, they were polished using a series of pastes, washed in alcohol and dried. Finally, the ingot sections were etched with Nital reagent for 5 min each. The microstructure observations were performed using a Keyence VHX–7000 (Keyence Corp., Osaka, Japan) light microscope (LM). The thickness of graphite particles was measured across their thicker part using DigitalMicrograph (GMS version 3.51.3720.0) software by Gatan Inc. (Pleasanton, CA, USA).

## 3. Results

### 3.1. Microstructure of DC and VC Alloy with High Al and Cr Additions

**Figure 4:**
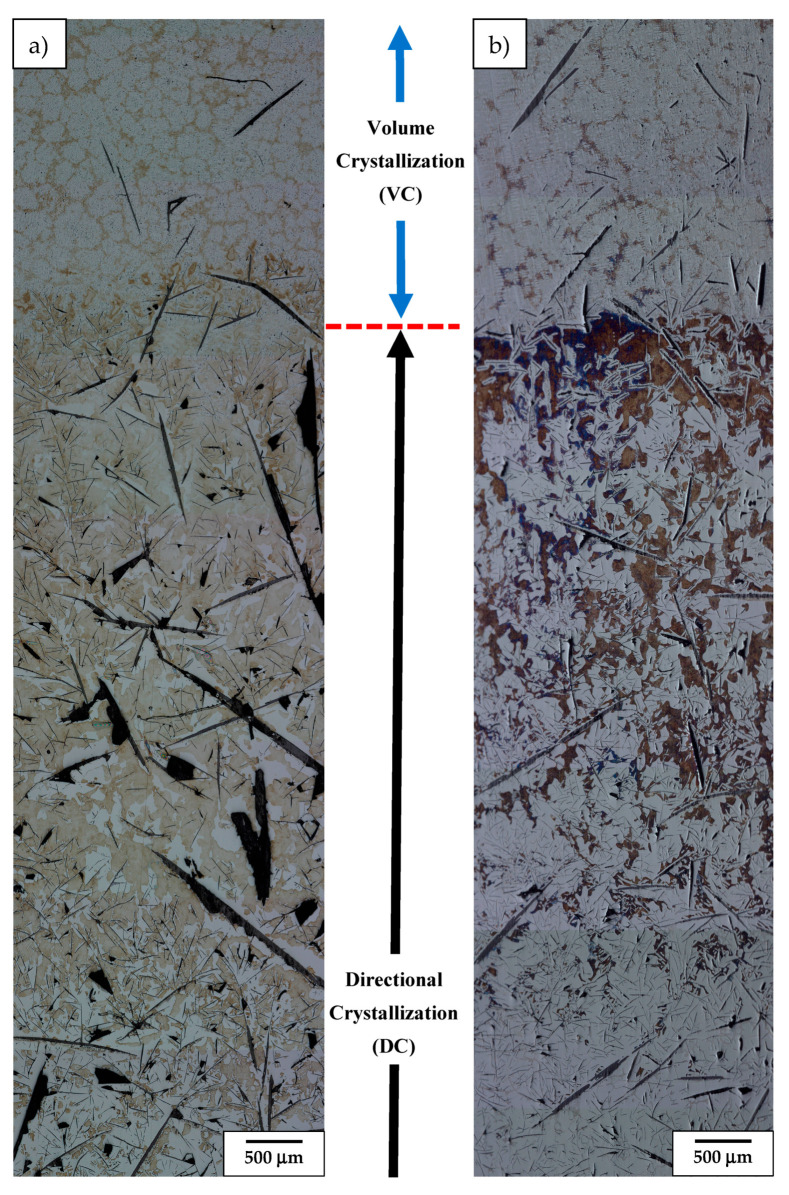
LM overview image of longitudinal section of Nital etched alloy 1 retracted at 5 mm/h (**a**) and 9 mm/h (**b**) and finally quenched.

The LM microstructure of alloy 1 (i.e., having the highest Al and Cr additions) was initially retracted from the hot area into a cooled section at a relatively slow (sample 1.1, 5 mm/h) and medium rate (sample 1.2, 9 mm/h), only to be finally quenched, as presented in Figure 4. This showed that the bottom part of such obtained ingots (the area first subjected to directional crystallization, also called a “transition zone” [26]) is dominated by a haphazardly oriented mixture of coarse and fine graphite nodules immersed in the ferritic matrix. In the slowly cooled sample, that part of the ingot also carried some pearlite colonies, while the bottom of the more rapidly cooled one was practically free of them. Moving towards the middle section of these ingots, a number of thick graphite nodules increases on account of the thinner ones (part of stable directional crystallization (DC) [26]). Additionally, the matrix of the ingots in these areas turned out to be partly of the perlite type, forming a major and minor phase fraction in the faster and slower cooled ingots, respectively. The upper part of the ingots’ presented microstructure developed after the ingots were thrown into the cooling medium, i.e., after volume crystallization (VC). It was in both cases dominated by a cell-like structure with the only occasional presence of thick, elongated graphite nodules.

**Figure 5:**
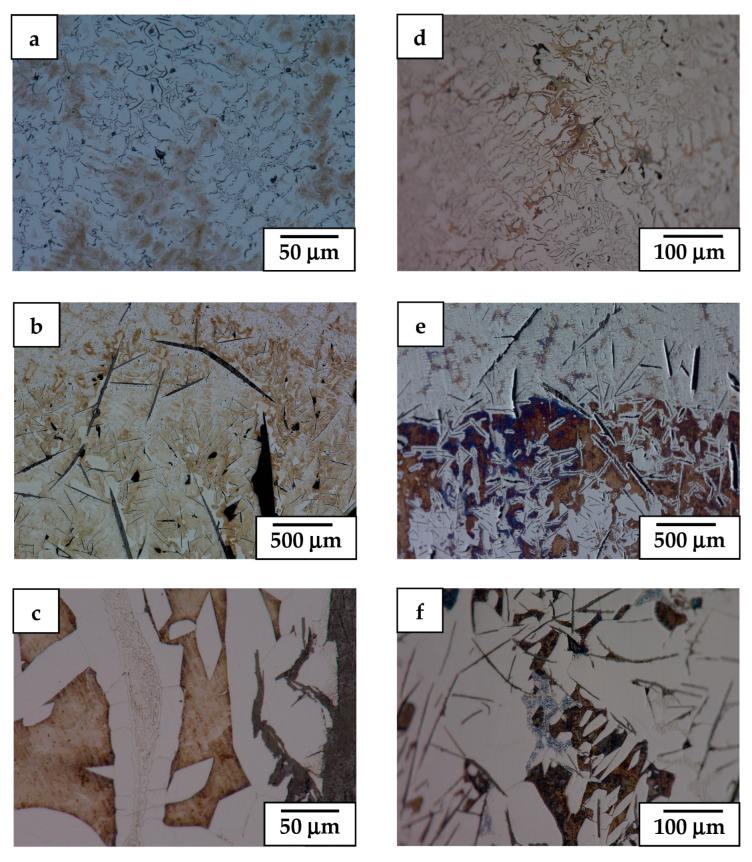
LM image of alloy 1 presenting microstructure details of sample retracted at 5 mm/h (**a**–**c**) and 9 mm/h (**d**–**f**) and finally quenched.

More detailed LM observation at higher magnification of these ingots (samples: 1.1, 1.2) is presented in Figure 5. It shows that at the area of the VC, the sides of graphite nodules as well as in between some of the perlite islands experienced precipitation of carbide eutectic (Figure 5c,f). At the boundary between the DC and the VC areas of slowly retracted sample 1.1, the pearlite colonies were substituted by a fully ferritic matrix exhibiting dendritic segregation of introduced alloying additions (revealed by etching) (Figure 5b). In the case of the faster retracted sample 1.2, the switch from controlled cooling conditions into quenching was evidenced by a rapid increase in the amount of pearlite up to the point at which the ferrite remained only in the form of thin envelops surrounding fine graphite flakes (Figure 5e). The VC areas of both ingots presented very similar microstructures characterized by the presence of elliptical islands of ferrite phase (slashed dendrite arms) surrounded by channels filled with eutectic formed by fine twisted graphite flakes in a ferritic matrix, i.e., chunky graphite [27] (Figure 5a,d). Aside of that, the occasional presence of coarse compacted graphite particles was noted. Additionally, segregation of alloying additions (Figure 4a) as well as accompanying precipitation of carbides and remnants of small perlite colonies (Figure 4b) contributed to the formation of branches with a cell-like structure.

### 3.2. Microstructure of DC and VC Alloy with Medium Al and Cr Additions

**Figure 6:**
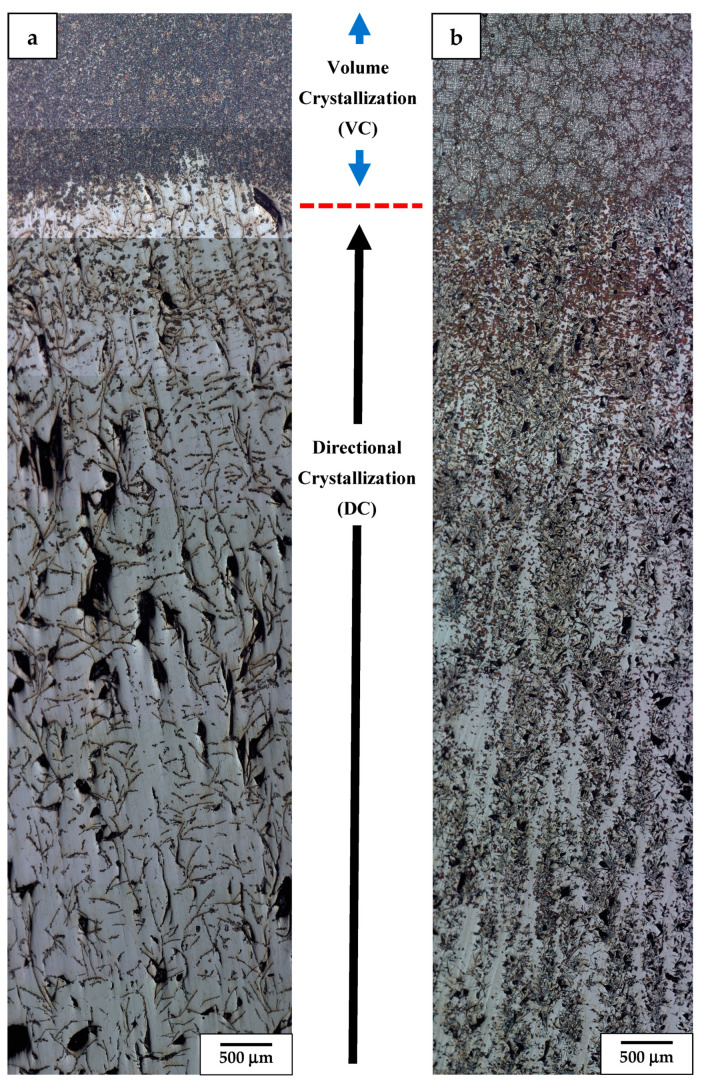
LM overview image of longitudinal section of Nital etched alloy 2 retracted at 9 mm/h (**a**) and 30 mm/h (**b**) and finally quenched.

The LM microstructures of alloy 2 (having roughly halved Al and Cr additions), which was retracted from the hot area into a cooled section at a medium (sample 2.1, 9 mm/h) and high rate (sample 2.2, 30 mm/h) only to be finally quenched, are presented in Figure 6. The phase content and microstructure characteristics quite closely follow the one observed in sample 1.2 (9 mm/h and quenched), i.e., showed just two zones. Especially, the bottom and middle part (DC) of sample 2.1 demonstrated a comparable amount of coarse and fine compacted graphite particles even as in this case they were losing their lath shape on account of compacted ones (Figure 6a). Increasing the retraction rate (sample 2.2) resulted in strong refinement of the graphite particles with a simultaneous increase in their density (Figure 6b). This was accompanied by the development of bands of whitish ferrite phase with jagged sides, elongated along the retraction direction (ingot long axis). The presence of perlite started to show up in larger amounts only close to and at the DC/VC boundary. The upper part of the castings showed a microstructure developed during quenching (VC) of this alloy. In both cases, it was highly refined and free of larger graphite nodules. However, an evident cell-like structure in the VC zone developed only in the more quickly retracted sample 2.2, while the one obtained at a medium velocity carried no sign of them.

**Figure 7:**
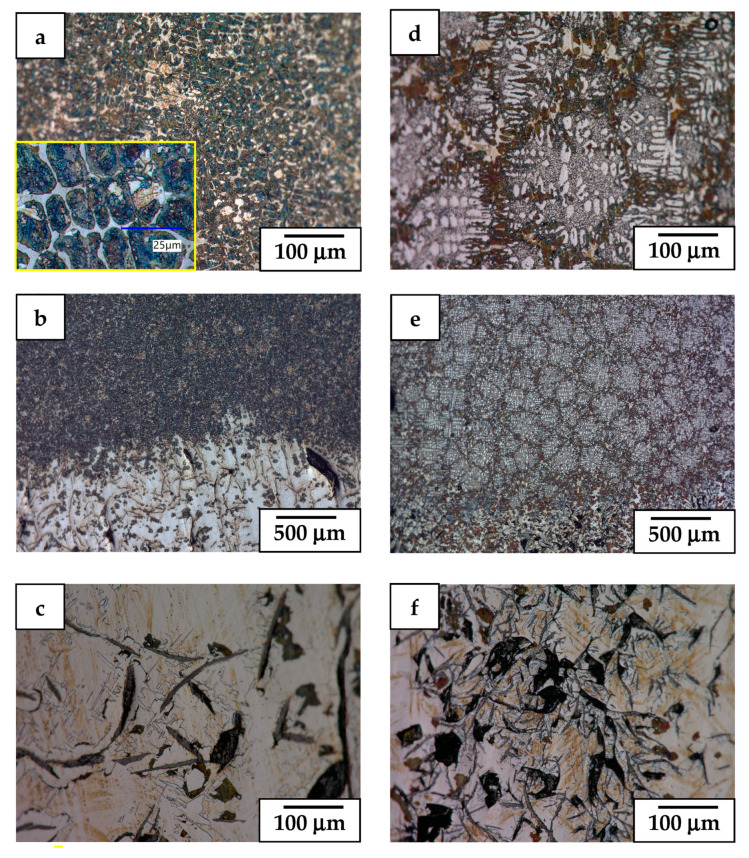
LM image of alloy 2 presenting microstructure details of sample retracted at 9 mm/h (**a**–**c**) and 30 mm/h (**d**–**f**) and finally quenched. Magnified pearlite islands surrounded by carbides are presented in (**a**).

The LM observation at higher magnification of the DC zone in sample 2.1 (Figure 7) revealed that the graphite nodules were frequently enveloped with blocky or lath-like ferrite grains (Figure 7c). Only in the upper part of this zone was the presence of small areas of pearlite noted. Otherwise, the bottom part (DC) of this sample was filled with ausferrite, evidenced by its yellowish color. The perlite in this zone appeared only in the upper part of the DC zone in the form of small dark brown-bluish islands. The DC zone in sample 2.2 was similar to that in sample 2.1, with the exception that the graphite nodules were refined and the number of perlite islands was significantly increased (Figure 7f). Additionally, the ausferrite was characterized by an acicular character (imitating martensite). The boundary between the DC and VC zones in both samples carried, as in case of alloy 1, a larger volume of perlite phase (Figure 7b,e). The fast-quenched upper part of castings (VC) of this alloy again showed a highly refined microstructure like the previous alloy (Figure 7a,d). Especially close similarities to the VC in samples 1.1 and 1.2 show that the structure formed in sample 2.2, i.e., with chains of ferritic islands surrounded by fine ferrite–graphite eutectic, includes a superimposed net of well-developed cells (Figure 7d). The cell walls consisted mostly of small perlite colonies and a much lesser number of elongated carbides. On the other hand, the VC zone in sample 2.1 (quenched in same way as sample 2.2) was found to be built predominantly of colonies of pearlite filling in the former austenite dendrite arms with the carbides and ferrite grains filling in the remaining free space (Figure 7a). The latter two phases also tended to form a net of cell walls, but it was both less developed and masked by perlite dark contrast.

### 3.3. Microstructure of DC and VC Alloy with Medium Al Addition and Cr Free

**Figure 8:**
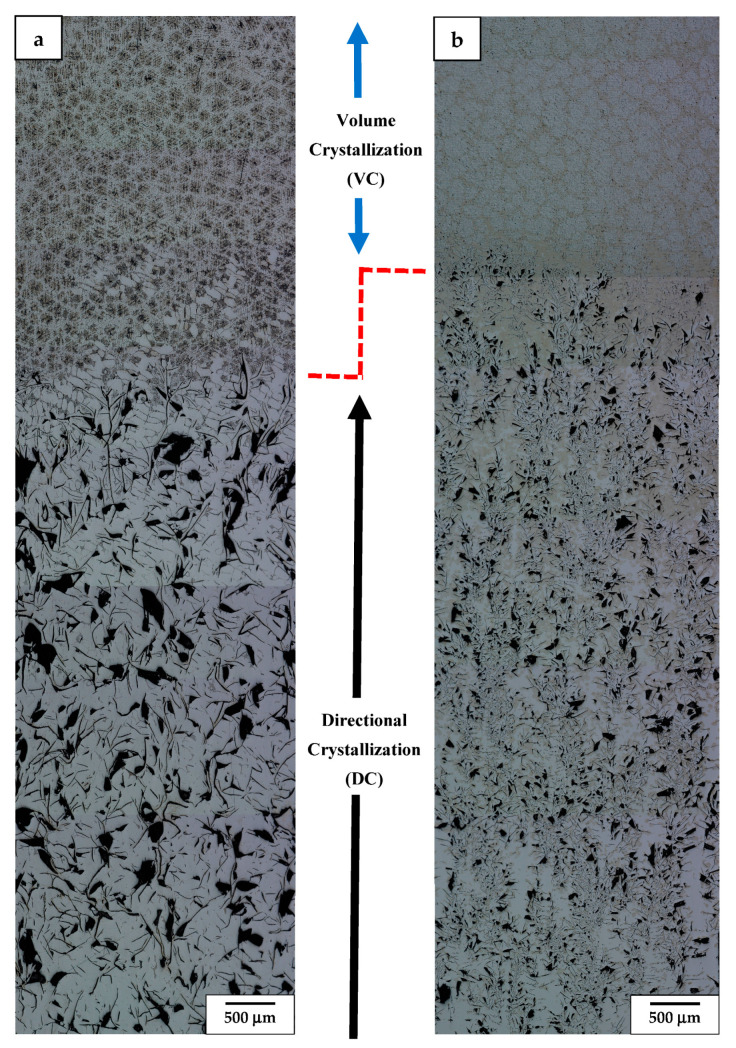
LM overview image of longitudinal section of Nital etched alloy 3 retracted at 9 mm/h using crucible with *ϕ*—5 mm (**a**) and *ϕ*—7 mm (**b**) and finally quenched.

The LM observation of casting of alloy 3 (with the same Al addition as alloy 2, i.e., half of alloy 1, but practically no Cr), which was retracted from the hot area into a cooled section at the same medium rate (9 mm/h) but crystallized in tubes with an inner diameter of *ϕ*—5 mm (3.1) and *ϕ*—7 mm (3.2), respectively, is presented in Figure 8. The results showed that using a tubular crucible with the same diameter resulted in precipitation of coarser graphite nodular particles in the DC zone, like in sample 2.1. On the other hand, the DC material crystallized in a tube with a larger diameter was characterized by more refined graphite particles, i.e., the coarse ones of that phase were practically absent. Simultaneously, judging the etching effects, the latter casting seemed to be formed of two phases, i.e., whitish and light yellowish. The microstructure of the quenched VC zone in both castings consisted of a highly refined microstructure with a superimposed cellular structure. The cells’ average dimension was at least double the size in the case of the latter (3.2) as compared with the former (3.1).

**Figure 9:**
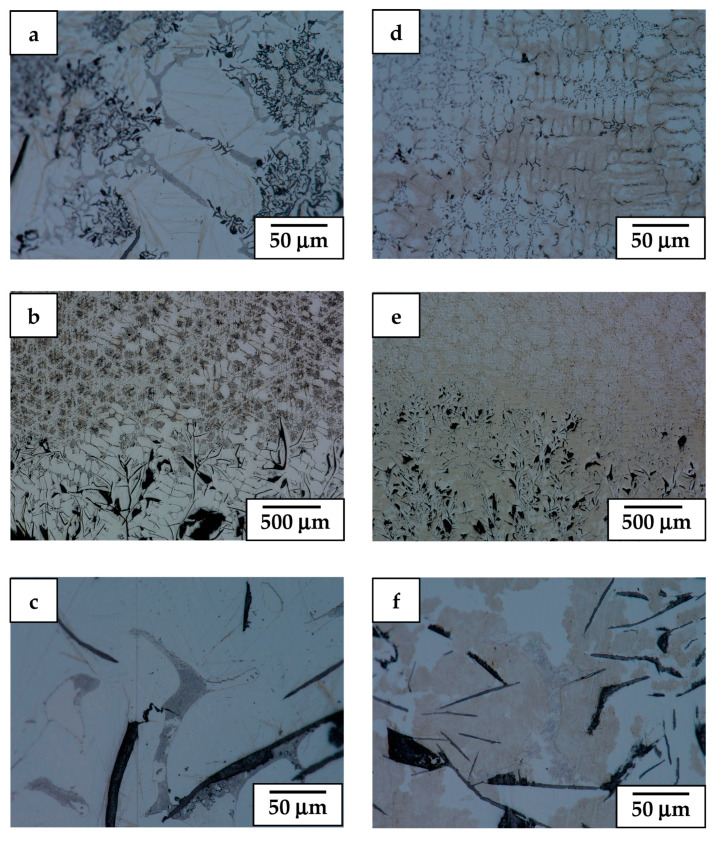
LM image of alloy 3 presenting microstructure details of sample retracted at 9 mm/h using tube of *ϕ*—5 mm (**a**–**c**) and *ϕ*—7 mm (**d**–**f**) and finally quenched.

The LM observation at a higher magnification of the DC zone in casting of *ϕ*—5 mm (sample 3.1, Figure 9) showed that the graphite particles were surrounded by ferrite grains (Figure 9c). Occasionally, the presence of martensite needles nucleated at graphite boundaries was also noted. The carbides were found to precipitate both at graphite particles as well as within the ferrite areas. The DC zone in casting of the alloy with the same composition but with a larger diameter, i.e., 7 mm (sample 3.2), was characterized by the presence of numerous refined carbon flakes and an even more noticeable appearance of alloyed ferrite occupying most of the graphite boundaries (Figure 9f). The boundaries between DC and VC zones showed some similarities, such as a strongly corrugated dividing line (Figure 9b,e). The upper part of the castings representing the VC zones were filled with elliptical fine ferrite grains separated by agglomerates of very fine carbon-fluff-type graphite flakes (chunky graphite) and some carbides (Figure 9a,d). In the case of sample 3.1, the ferrite grains in this zone usually carried a number of martensite needles (Figure 9a), while the thicker casting of the same alloy was free of this phase (Figure 9d). The VC zone in the latter casting also showed a cellular structure attributed to the segregation of alloying additions and precipitation of carbides.

### 3.4. Assessment of Graphite Morphology in Investigated Alloys

**Figure 10:**
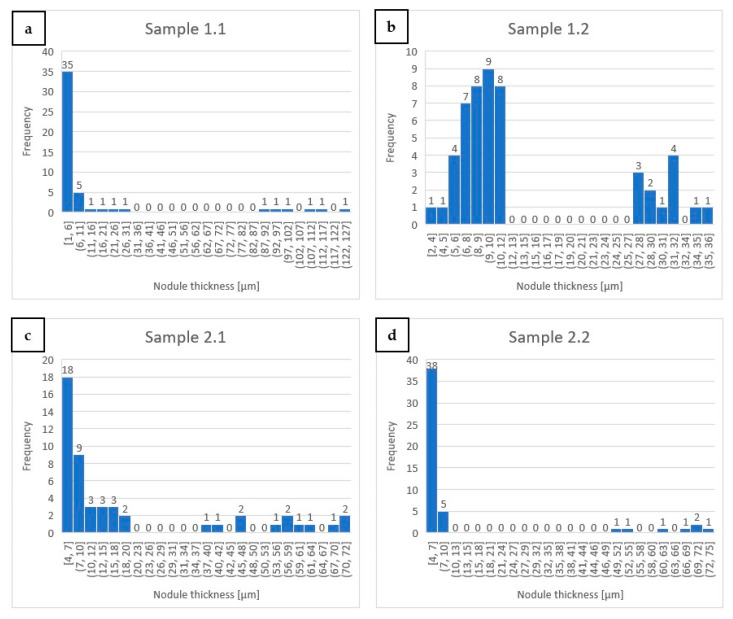
Plots presenting distribution of maximum thickness of graphite particles (flakes, laths and others) in the DC parts of 1.1 (**a**), 1.2 (**b**), 2.1 (**c**) and 2.2 (**d**) alloys.

The graphite precipitates in all investigated alloys showed a bi-modal size distribution, i.e., they precipitated both in the form of coarse primary lath-like or compacted graphite particles as well as highly refined needles or flakes. The primary graphite particles preceding precipitation of the austenite grains in samples 1.1 and 1.2 (both high aluminum with retraction rates of 5 mm/h and 9 mm/h, respectively) formed strongly elongated laths, but the thickness of the former sample was nearly twice that of the latter (Figure 10a,b). The primary graphite particles in the casting of alloys 2.1 and 2.2 (having nearly halved Al and strongly lowered Cr addition, with retraction rates of 9 mm/h and 30 mm/h, respectively) changed to more bulky ones, but again, the former had grown to a much larger size than the latter (Figure 10c,d). The thickness distribution of secondary graphite particles present in the form of thin flakes closely followed the above relationship, i.e., the raised retraction caused diminishing of the flakes’ thickness.

## 4. Discussion

The experiment was based on the controlled retraction of samples melted in thin ceramic tubes from the hot section into the cold section of a purpose-built special stand, as in the Bridgman–Stockbarger method [12]. It allowed the achievement of directional crystallization of SiMo5-1-based alloys having from ~1.5 to ~3.5 wt. % Al and from ~1.0 to ~2.5 wt. % Cr. Their prolonged exposure to high temperature in the furnace must have resulted in the removal of Mg. This explains a total decay of spheroidization tendency, which has been observed in master alloys serving as a source of samples in other experiments [26]. However, with real castings, even as the crystallization front propagates toward their core at around presently applied cooling rates, this element (Mg) would be trapped inside [5,28], especially as it is introduced into the melt just before casting and, in such a case, the spherical shape of the graphite nodules is retained. This means that the size distribution of graphite nodules as well as their location in reference to other microstructure components should also be maintained in slowly cooled commercial heavy castings.

The graphite in the DC zone formed larger nodules at lower retraction rates, controlling the velocity of the crystallization front. Increasing retraction rates resulted in refinement of the graphite particles, which was especially noticeable in the case of the largest ones in respective castings, i.e., in 1.1 (5 mm/h) as compared with 1.2 (9 mm/h) and in 2.1 (9 mm/h) as compared with 2.2 (30 mm/h). The data published on similarly crystallized alloys with 3.65–3.94% C, 1.15–2.04% Si, ~0.6% Mn, ~0.2% Cr and others <0.1% showed same relationship concerning microstructure refining level vs. crystallization velocity [26]. The other factor strongly affecting the microstructure refinement was the rate of heat dissipation that occurred during the transfer of the tubular crucible from the hot section to the cold section of the used apparatus. In the scenario where the same retraction rate denotes the progress of the crystallization front but at a much steeper temperature gradient, the graphite particles were much smaller in the crucible with a thinner wall.

The other characteristic feature of the presently obtained castings was the bi-modal size distribution of the graphite particles. This indicates that the graphite lath-like particles are the first to be nucleated in molten alloy and undertake fast growth due to rapid diffusion of C atoms in liquid. This agrees with the hyper-eutectoid character of these alloys, i.e., in accordance with their respective CE ranging from 4.75 to 5.13, calculated using Formula (1). However, at a certain temperature, the nucleation of austenite starts at graphite facets, cutting off easy access of the remaining C atoms in the liquid. This process is similar to the formation of bull’s eyes in the spheroidized cast iron [29]. In the next stage, the growth of the austenite grains contributes to oversaturating the remaining melt in carbon enforcing nucleation of new graphite flakes in between the expanding austenitic grains. The first stage of this process corresponds to a uninodular (UN) crystallization model, while the second one corresponds to a multinodular (MN) model [30]. In the case of a higher velocity of oriented crystallization front growth, such as 30 mm/h applied in the case of 2.2 casting, the austenite grains are significantly extended along the direction of their progress accompanied by parallel bands of secondary fine graphite flakes located in between. That type of crystallization bears close resemblance to that obtained during directional crystallization of grey iron with 3.86% C, 1.67% Si, and others <0.5, but at a much lower retraction rate (5 μm/s) [31].

The presently applied intermediate Al and Cr additions into SiMo-type DCI (alloy 3: 1.48% Al–0.09% Cr; and alloy 2: 1.87Al–1.01% Cr), which were subjected to directional crystallization at a 9 mm/h retraction rate, showed the presence of a significant amount of coarse compact and vermicular graphite, i.e., like in other SiMo-type alloys with Al addition [19,20,24]. Increasing the retraction rate, as in the case of sample 2.1, from 9 mm/h to 30 mm/h caused significant refinement of graphite particles. However, in samples (1.1 and 1.2) of the alloy with higher Cr and Al (3.50% Al–2.43% Cr) contents, within the DC area, the graphite precipitates of an altogether different shape and size, as compared to the alloy with lower alloying additions, were formed. This agrees with the literature data documenting that Al not only has pro-graphitizing properties but also acts as a strong anti-spheroidizer [24,25,32]. According to ASTM A247, their shape was the closest to the VII-type in all samples [27,33,34]. Regarding the spatial distribution, graphite type C showed the best match with that represented by samples 1.1 and 1.2, while graphite type A was closest for others. As for the VC area, the graphite precipitates were of the VII-type shape in samples 1.1 and 1.2, while they had an IV-type shape in the other classes. The most spectacular effect of the highest introduced alloying additions was a change in graphite particle shape, which was especially eye-catching in the case of the largest one, i.e., from a compacted type to a lath-like structure. This could only be attributed to the more than doubling of Al addition as Cr is tied up in pearlite formation, i.e., in Fe_3_C-type carbides [20].

**Figure 11:**
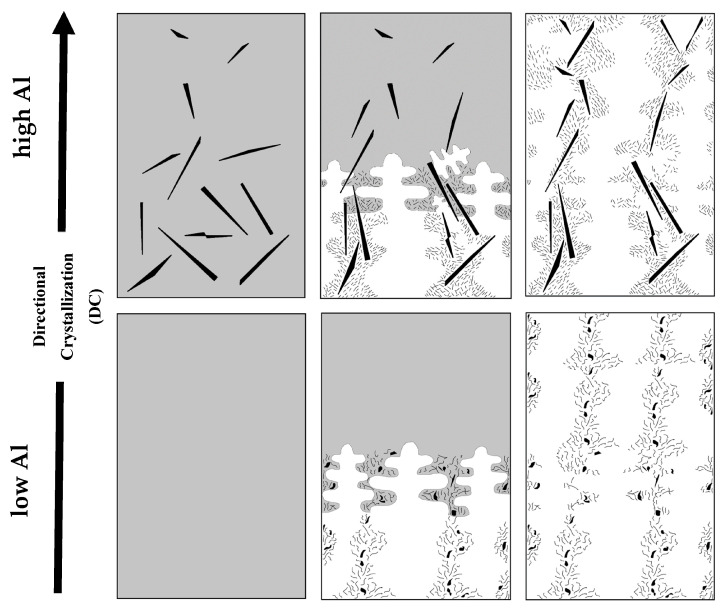
Scheme presenting progress of directional crystallization of SiMo-type cast iron modified with low (1.5–2%) and high (3.5%) Al additions; grey; melt, black; graphite and white; austenite.

The enforced directional crystallization evidenced itself most clearly in the alloy with lower applied alloying additions and retracted at relatively high rates of 9 mm/h and 30 mm/h (samples 3.2 and 2.2), which presented bands of ferrite extending along or at least at an acute angle to the tubular crucible long axis (samples 3.3, 3.2 and 2.2). This showed a close resemblance to a stable zone in the directionally crystallized cast iron with up to 2% of Si as a main alloying addition [31]. Rising of the Al addition in alloy 1 up to ~3.5% resulted in accelerated nucleation and the growth of large randomly oriented laths of graphite extending far ahead of crystallization front. This mechanism evidenced itself by the presence of the same large graphite laths in the VC zone. Roughly the same number of this precipitate meant that at that level of Al addition and with applied retraction rates, the graphite flotation was avoided. The cooling down of the melt filled with large graphite laths during its withdrawal from the hot section restricted the growth of extended dendrite-like austenite grains, which first had to adjust to available free space in the rest of the melt and only then expand along the crucible axis. The raised level of Cr caused precipitation of perlite islands masking the original austenite grain arrangement. However, the fine graphite flakes precipitating in between the arms of austenite dendrites with the progress of crystallization allowed the original arrangement of crystallites of these phases to be visualized. The schematic presentation of the effect of a raised Al level on the directional crystallization mechanism is given in Figure 11. Practically the same deviation of the long axis of austenite dendrites was documented in previously mentioned directionally crystallized cast iron with up to ~2 at. % Si as the main alloying addition. In that case, the obstacles were the loose agglomeration of fine graphite flakes nucleated by the Ce inoculant.

## 5. Summary and Conclusions

The experiments with directional crystallization of SiMo DCIs with up to ~3.5% Al and 2.4% Cr using the Bridgman–Stockbarger method showed that their phase composition and microstructure were highly sensitive to both the type and level of introduced alloying additions. On the other hand, the used rate of progress of the crystallization front from 5 mm/h to 9 mm/h and to 30 mm/h were more instrumental in microstructure refinement. It was significant that under the applied experimental conditions—specifically, having a tubular crucible outside diameter <10 mm and lowering the wall thickness from 3 mm to 1 mm—the temperature gradient at the crystallization front increased,. It caused refining of the microstructure by a factor comparable to at least doubling the retraction rate. This strong microstructure refinement took place even as, in the crucible with the thinner wall, the amount of crystallizing material was much higher. The performed experiments allowed the following conclusions to be drawn:

- Alloying SiMo-type alloy with ~1% to ~2% of Al caused precipitation of compacted graphite particles as well as thin flake ones. Increasing the Al addition up to 3.5% replaced the compacted graphite with lath-like particles, but flake ones remained unaffected. The precipitation of large compacted and lath-like graphite preceded the crystallization of austenite, while flakes followed it.
- Alloying SiMo-type alloy with 1% to 2.4% of Cr resulted in the formation of a low and high content of pearlite, respectively. The post-solidification transformation started with precipitation of ferrite grains at the graphite particles or boundaries of carbide colonies, while pearlite islands nucleated at ferrite grain boundaries.
- The increased directional crystallization rate of the SiMo-type alloy with intermediate Al additions of ~2% caused formation of a mixed microstructure, where the Widmanstätten and ausferrite phases were substituted with the latter phase. The same action with the alloys having increased both the Al and Cr levels resulted in refinement of the flake graphite as well as an increase in the amount of ferrite at the expanse of pearlite.
- The switch from directional crystallization to quenching causes the formation of cells of walls enriched in alloying additions and insides filled with chunky graphite. This means that the transition from a liquid to solid state starts with nucleation of islands of fine austenite dendrites with austenite–graphite eutectic separating them. As they expand, they push alloying addition to its sides, promoting carbide or pearlite formation in these places.

## Figures and Tables

**Table 1:** Chemical composition (wt. %) of alloys and crucible retraction velocities v (mm/h).

Alloy	Sample No.	v (mm/h)	Crucible	C	Si	Mo	Al	Cr	P	S	Ti	Mn	Cu	Ni	Fe
1	1.1	5	SiO_2_ *ϕ*—5 mm	3.07	5.43	0.94	3.50	2.43	0.03	0.02	0.02	0.43	0.08	0.05	rest
	1.2	9													
2	2.1	9	Al_2_O_3_ thick * ϕ—5 mm	2.60	5.52	1.02	1.87	1.01	0.05	0.01	0.02	0.46	0.12	0.06	rest
	2.2	30													
3	3.1	9	Al_2_O_3_ thick * ϕ—5 mm	3.13	5.27	0.78	1.48	0.09	0.03	0.01	0.02	0.46	0.10	0.02	rest
	3.2	9	Al_2_O_3_ thin * ϕ—7 mm												

* Thick and thin denote crucible wall type.

**Table 2:** CE, S_c_, T_st_ and T_met_ values of tested alloys.

Alloy	CE	S_c_	T_st_ (°C)	T_met_ (°C)
1	5.13	1.39	1203	1015
2	4.92	1.27	1190	1038
3	4.75	1.19	1186	1048

## Data Availability

Data are contained within the article.
